# Lexical alignment in triadic communication

**DOI:** 10.3389/fpsyg.2015.00127

**Published:** 2015-02-13

**Authors:** Anouschka Foltz, Judith Gaspers, Kristina Thiele, Prisca Stenneken, Philipp Cimiano

**Affiliations:** ^1^Cognitive Interaction Technology – Center of Excellence, Bielefeld UniversityBielefeld, Germany; ^2^Collaborative Research Center “Alignment in Communication,” Bielefeld UniversityBielefeld, German; ^3^School of Linguistics & English Language, Bangor UniversityBangor, UK; ^4^Department of Special Education and Rehabilitation, Faculty of Human Sciences, University of CologneCologne, Germany

**Keywords:** lexical alignment, communication, non-addressee-centered behavior, addressee-centered behavior, priming

## Abstract

Lexical alignment refers to the adoption of one’s interlocutor’s lexical items. Accounts of the mechanisms underlying such lexical alignment differ (among other aspects) in the role assigned to addressee-centered behavior. In this study, we used a triadic communicative situation to test which factors may modulate the extent to which participants’ lexical alignment reflects addressee-centered behavior. Pairs of naïve participants played a picture matching game and received information about the order in which pictures were to be matched from a voice over headphones. On critical trials, participants did or did not hear a name for the picture to be matched next over headphones. Importantly, when the voice over headphones provided a name, it did not match the name that the interlocutor had previously used to describe the object. Participants overwhelmingly used the word that the voice over headphones provided. This result points to non-addressee-centered behavior and is discussed in terms of disrupting alignment with the interlocutor as well as in terms of establishing alignment with the voice over headphones. In addition, the type of picture (line drawing vs. tangram shape) independently modulated lexical alignment, such that participants showed more lexical alignment to their interlocutor for (more ambiguous) tangram shapes compared to line drawings. Overall, the results point to a rather large role for non-addressee-centered behavior during lexical alignment.

## INTRODUCTION

During conversation, speakers tend to adopt a variety of aspects of their interlocutors’ linguistic and non-linguistic behavior. For example, speakers may adopt their interlocutor’s syntactic structures (Branigan et al., 2000), lexical items (e.g., Brennan and Clark, 1996), description schemes (Garrod and Anderson, 1987), word pronunciation (Pardo, 2006), body posture (Chartrand and Bargh, 1999), etc. Such alignment of interlocutors is pervasive in communication (Wachsmuth et al., 2013) and has been suggested to contribute to communicative success (Pickering and Garrod, 2006).

Despite the pervasiveness and potential importance of alignment for communication, there is no unified account of the mechanisms underlying this phenomenon. This paper focuses on lexical alignment. A number of different theoretical accounts have attempted to spell out the mechanisms that lead to lexical alignment in conversation (e.g., Brennan and Clark, 1996; Pickering and Garrod, 2004; Keysar, 2007). These accounts differ, among other things, in the extent to which utterances are assumed to be designed for a particular interlocutor.

Keysar’s (2007) egocentric processing approach, for example, proposes that the speaker’s own perspective is dominant and that behavior that takes the listener into account plays a minor role. Speakers may then adopt their interlocutor’s lexical items etc. because this is easier from a production standpoint (for example, because these lexical items had been heard most recently and may thus be pre-activated), not because adopting lexical items may be helpful for the listener. Similarly, Pickering and Garrod’s (2004, see also Garrod and Pickering, 2006) prominent interactive alignment model assumes that alignment is largely the result of automatic lower level priming and that strategic behavior, such as designing one’s utterance with the interlocutor in mind, occurs only as a repair, i.e., when a misunderstanding has occurred and needs to be attended to. In particular, they argue that such strategic processes occur on top of a basic and automatic tendency to align (Garrod and Pickering, 2007).

Other accounts of lexical alignment highlight strategic processes and assign a larger role to the interlocutor. For example, a conceptual pacts account (Brennan and Clark, 1996) assumes that interlocutors choose referring expressions based on so-called conceptual pacts that they have formed with the particular interlocutor during a conversation. These conceptual pacts arise from previous choices made during a conversation and are thus temporary and flexible. Similarly, a communicative design account (Clark, 1996; see also Branigan et al., 2011) assumes that speakers design their utterances for specific speakers.

There is ample evidence in the literature that lexical alignment has a considerable partner-specific component, such that participants align to the particular individual with whom they are interacting. Thus, speakers lexically align to a specific listener. For example, Brennan and Clark (1996) found evidence that the conversational history with a particular interlocutor affects lexical choices. In particular, interlocutors in the study referred to a picture of a certain kind of shoe as a *shoe* when the task involved only one item of that category. When the task then involved pictures of three different kinds of shoes, interlocutors switched to more specific terms, such as *pennyloafer*, to describe individual shoes. Finally, when the task returned to involving only one picture of a shoe, participants kept referring to this shoe as a *pennyloafer*, even though a more basic-level term would have sufficed. In addition, participants were more likely to keep using the term *pennyloafer* and less likely to return to using the basic-level term *shoe* with the same interlocutor compared to a new interlocutor.

In addition, Metzing and Brennan’s (2003) eyetracking study found evidence that listeners encode partner-specific lexical information during a conversation. In their study, a participant played a matching task with a confederate partner who, for example, used the expression *the shiny cylinder* to refer to a certain object. Later either the original partner or a new partner referred to the same object either as *the shiny cylinder* or as *the silver pipe*. Participants looked at the target object significantly later when the original partner used the term *the silver pipe* compared to when the new partner used the term *the silver pipe*. In contrast, participants looked at the target object equally fast when the original partner used the term *the shiny cylinder* compared to when the new partner used this term. This suggests that listeners expect their conversational partners to continue using the lexical items that have been established during the conversation. In addition, suddenly switching referring expressions within a conversation results in significant processing cost.

In addition to partner-specific effects concerning individual partners, a number of studies found that certain properties assigned to a partner, such as competence or status, may affect lexical alignment. Here, lexical alignment is not adapted to specific partners, but to a general or stereotypical partner and the properties assigned to this kind of partner. For example, Branigan et al. (2011) found that the degree of lexical alignment is related to an interlocutor’s beliefs about their conversation partner’s (linguistic) competence: participants showed stronger lexical alignment when they believed they interacted with a computer compared to a human, and stronger lexical alignment when they believed they interacted with a less capable compared to a more capable computer. These differences in alignment occurred even though the actual linguistic behavior of the interlocutor was kept constant. Furthermore, Pirie (2010) found marginally higher alignment to the interlocutor if participants believed that she was a professor compared to a fellow university student.

A recent study on syntactic alignment investigated to what extent alignment may be socially mediated. Participants in Weatherholtz et al. (2014) listened to a speaker before completing a picture description task to assess their syntactic alignment to the speaker. They found that alignment was influenced by how standard participants considered the speaker’s accent to be, by how similar to the speaker they perceived themselves to be, and by participants’ conflict management styles. Here again, certain properties assigned to a person (accent standardness and similarity) influenced the participants’ degree of alignment. In addition, individual differences in the participants themselves (conflict management style) influenced the degree of alignment.

Jucks et al. (2008) used an experimental design that introduced two sources of input, only one of which was the interlocutor, to explore to what extent lexical choices may be designed for specific or general partners. Medical students responded to an email inquiry from a patient. To aid them in their response, they were presented with a flow chart. The patient’s email as well as the flow chart contained medical terms in either technical or everyday language. If both sources of input contained the same medical terms (i.e., either in technical or in everyday language), participants adopted these medical terms in their responses. But if the sources of input differed in the type of medical terms used, then participants used both technical and everyday language, with a preference for everyday language, regardless of whether the patient or the flow chart had introduced the everyday language. In contrast to the dyadic studies mentioned above, this pattern of results suggests that participants did not adapt to a specific partner, but used all words available to them, regardless of whether or not they came from the partner. In addition, participants preferred everyday language over technical language when presented with both technical and everyday language suggesting that they adapted to a non-expert partner.

The present study also uses a design with two sources of input, only one of which is the actual interlocutor. In particular, the current experiment uses a triadic communicative situation to explore several factors that may modulate lexical alignment with a specific partner (cf. Behnel et al., 2013, who also looked at lexical alignment in triadic communication). Two naïve participants – a director and a matcher – played a matching game, but also received game-relevant information from a voice over headphones. Thus, there were two sources of input (the matcher and the voice over headphones). However, in our design only the matcher was the actual conversation partner, who the director needed to address and who needed to perform a task based on the director’s instructions. Thus, the director and the matcher performed a collaborative task together, whereas the voice over headphones was not involved in the actual conversation, but provided information to the director that allowed him or her to adhere to the rules of the collaborative task.

This triadic communicative situation allows us to set up situations where the conversation partner and the voice over headphones have previously provided different names for the same object. For example, the director’s conversation partner (matcher) had referred to an object in a picture as *jug* some trials ago. During the target trial, the voice over headphones informed the director that *next is the pitcher*. The director then informed the matcher where to place the picture of the jug/pitcher. We can then explore whether in these situations directors lexically align with their conversation partner (e.g., *put the jug in the top left*) or with the voice over headphones (e.g., *put the pitcher in the top left*).

To investigate factors that may modulate lexical alignment to a specific partner, the following experiment presents three experimental manipulations: first, participants were either presented with an alternative lexical item over headphones (e.g., *next is the pitcher*) or not (e.g., *next is the third item*). When presented with an alternative lexical item, participants could either align with their specific conversation partner (and form a conceptual pact) or adopt the word proposed by the voice over headphones. In this latter case, they would show non-addressee-centered behavior, which may be egocentric in the sense that the word proposed by the voice is the most available. Alternatively, they may strategically align with the voice over headphones rather than the actual conversation partner.

Second, participants either named line drawings or tangram shapes, which differ in that line drawings are less abstract and provide a smaller range of possible interpretations than tangram shapes. For example, a line drawing of a *pitcher* can only be interpreted as something semantically similar to a *pitcher*, for example, a *jug* or possibly even a *vase*. In contrast, tangram shapes can receive semantically unrelated interpretations. For example, the same tangram shape may be interpreted as either an *arrow* or a *rocket*. Thus, if the director does not align with the matcher, there is a greater risk of the matcher misunderstanding and selecting an incorrect picture in the case of tangram shapes compared to line drawings. If the director’s lexical choices are produced with the matcher’s needs in mind, then s/he should show stronger alignment to the matcher for tangram shapes compared to line drawings.

Third, there was either a long lag or a short lag between the interlocutor’s mention of a lexical item for a particular picture and mention of the alternative term over headphones. The lag between prime and target has been shown to affect lexical alignment in dyadic conversational situations. For example, Branigan et al. (2011) found less lexical alignment when eight filler pictures compared to when no filler pictures intervened between a human interlocutor’s mention of a dispreferred lexical item and the speaker’s lexical choice. We may find the same kind of lag effect in a triadic situation. In particular, directors may find it harder to remember the lexical item used by their interlocutor for longer compared to shorter lags. The alternative provided by the voice over headphones may then be a welcome recent alternative.

In addition to these experimental manipulations, we tested whether participants’ individual differences in picture naming ability and working memory capacity may affect the degree of lexical alignment to a specific partner. We tested participants’ (speeded) picture naming ability because the matching task involved picture naming: directors had to give each picture a name and then explain where the picture should be placed. Picture naming involves identifying the object, activating the name, and generating a response (cf. Johnson et al., 1996). In our experiment, participants saw the pictures before they needed to name them. Participants with good picture naming ability may be more likely to have already activated a particular name for a picture (possibly that used by their interlocutor earlier) before hearing the alternative word from the voice over headphones. Therefore, participants with better picture naming ability may be less susceptible to an alternative lexical item and thus show more alignment with their actual interlocutor than participants with poorer picture naming ability.

We tested participants’ working memory capacity because in our experiment aligning to the interlocutor involved remembering the name that the interlocutor had given a certain picture. In contrast, aligning to the voice over headphones involved re-using the most recently heard lexical item. Thus, alignment to the actual interlocutor may depend on working memory capacity, such that participants with better working memory capacity may show stronger lexical alignment to the actual interlocutor than participants with poorer working memory capacity. The idea that working memory may affect lexical alignment parallels proposals in the syntactic alignment literature (cf. Hartsuiker and Kolk, 1998; Foltz et al., 2014).

Finally, following an informal observation that participants who knew each other seemed to behave differently from participants who did not know each other, we also included level of acquaintance as a factor in the study. How the level of acquaintance may affect alignment with the actual conversation partner is an open question. Davis and Rusbult (2001) have shown that interlocutors who were dating showed more attitude alignment than interlocutors who did not know each other. On the other hand, Savitsky et al. (2011) found that participants showed more egocentric behavior when interacting with close friends compared to with strangers. Thus, it may be the case that pairs of participants who know each other well would show more alignment to each other than pairs who know each other less well or not at all, similar to attitude alignment. Alternatively, it may be the case that pairs of participants who know each other well would show more egocentric behavior than pairs who know each other less well or not at all. In our experimental paradigm, this would result in less alignment for pairs who know each other well compared to pairs who know each other less well or not at all.

## MATERIALS AND METHODS

### PARTICIPANTS

Forty pairs of adult native German-speakers participated in the study (18 male, 62 female, mean age = 26 years, SD = 5.3 years). All participants were students at Bielefeld University and received compensation for their participation. The research was approved by the ethics committee of the University Hospital Münster and conforms to the ethical standards of the Declaration of Helsinki. All participants gave informed consent to participate in the study.

### MATERIALS

The experiment has a 2 (alternative name) by 2 (picture type) by 2 (interlocutor mention – alternative name lag) design. The materials for this study consisted of pictures and audio recordings. There were two types of pictures: line drawings and tangram shapes. All experimental pictures occurred twice during the experiment, such that there was either a long or a short lag between successive naming of the same picture. Audio recordings were used for the alternative name manipulation. They either named (e.g., *next is the pitcher*) or did not name (e.g., *next is the third figure*) the object to be placed next.

Experimental pictures needed to fulfill two criteria: they should have two alternative names and both names should be common enough that participants may use them in the experiment. We therefore conducted a picture naming pilot study to select appropriate pictures and their associated alternative names. For the pilot study, eighty-seven participants provided names for a total of 62 pictures in an online naming study. Forty-two of the pictures were line drawings, taken either from the internet (17 pictures) or from Snodgrass and Vanderwart (1980, 25 pictures). Twenty pictures were tangram shapes, taken from Behnel et al. (2013). We included all pictures in the pilot study that were likely to elicit two alternative names: line drawings from Snodgrass and Vanderwart (1980) were included in our pilot study if they had a naming agreement of below 50% in Genzel et al.’s (1995) German standardization of the Snodgrass and Vanderwart (1980) pictures. Additional line drawings from the internet were included in the pilot study based on native speaker intuitions, i.e., if the authors suspected that a certain item may be given alternative names. To select tangram shapes for the pilot study, we inspected the raw data from Behnel et al.’s (2013) online elicitation study. Tangram shapes were included in the pilot study if the raw data included two frequently occurring referential expressions. Participants in the pilot study were instructed to name the pictures with just one word. This was done to encourage participants to provide a name for what they saw in the pictures and to prevent participants from giving detailed picture descriptions.

Based on the results from the pilot study, we selected eight line drawings and eight tangram shapes as experimental pictures for our study. These pictures received two different names with neither name being very strongly preferred or very strongly dispreferred. The two most frequently assigned names for the selected line drawings and tangram shapes are given in **Table 1**. The most frequent names of the selected line drawings were assigned on average by 58.5% of participants; the second most frequent names were assigned on average by 24.3% of participants. The remaining participants (on average 17.2%) assigned other names to the line drawings. Thus, most participants assigned one of two alternative names to the line drawings. Even though one of the alternative names was typically preferred, there was no very strong bias toward one alternative. The most frequent names for tangram shapes were assigned on average by 20.8% of participants; the second most frequent names were assigned on average by 15.7% of participants. The remaining participants (on average 63.5%) chose other names. Here, most participants assigned various other names to the tangram shapes. Thus, neither the most preferred, nor the second most preferred names were commonly chosen. This is not surprising since the tangram shapes are abstract and can be interpreted in many different ways. Importantly, no names were strongly preferred.

**Table 1:** The two most frequently assigned names for the line drawings and tangram shapes included in the study.

Line drawings		Tangram shapes	
Most frequent name	Second most frequent name	Most frequent name	Second most frequent name
Haare (hair, 55.2%)	Frisur (haircut, 23.0%)	Tisch (table, 33.3%)	Tor (gate, 23.0%)
Jacke (jacket, 57.5%)	Hemd (shirt, 21.8%)	Tänzer (dancer, 25.3%)	Mensch (human, 19.5%)
Flügel (grand piano, 77.0%)	Klavier (piano, 17.3%)	M (letter m, 16.1%)	Brücke (bridge, 13.8%)
Kanne (pitcher, 52.8%)	Krug (jug, 23.0%)	Kopf (head, 24.1%)	Sprechblase (speech bubble, 12.6%)
Robbe (seal, 43.6%)	Seehund (seal, 27.6%)	Kerze (candle, 13.8%)	Brunnen (fountain, 8.0%)
Möhre (carrot, 61.0%)	Karotte (carrot, 28.7%)	Teufel (devil, 19.5%)	Maske (mask, 19.5%)
Portemonnaie (wallet, 46.0%)	Geldbörse (wallet, 29.9%)	Pfeil (arrow, 15.0%)	Rakete (rocket, 12.6%)
Krawatte (tie, 74.7%)	Schlips (tie, 23.0%)	Wasserhahn (faucet, 19.5%)	Ofen (furnace, 16.1%)

*Translations and the percentages of participants who assigned the names are given in parentheses.*

Filler pictures should receive one strongly preferred name. We used data from previous studies to select filler pictures: selected filler line drawings and tangram shapes were described with one highly preferred term in Genzel et al. (1995) and Behnel et al. (2013), respectively. The filler line drawings depicted a snail, a bottle, an elephant, a dog, a comb, a ladder, and a sun. Filler tangram shapes were most commonly described as swan, Native American, rabbit, legs, fish, lamb, and mountains. Altogether, participants experienced eight trials with a line drawing filler picture, eight with a tangram shape filler picture, sixteen with a line drawing experimental picture, and sixteen with a tangram shape experimental picture.

**FIGURE 1:**
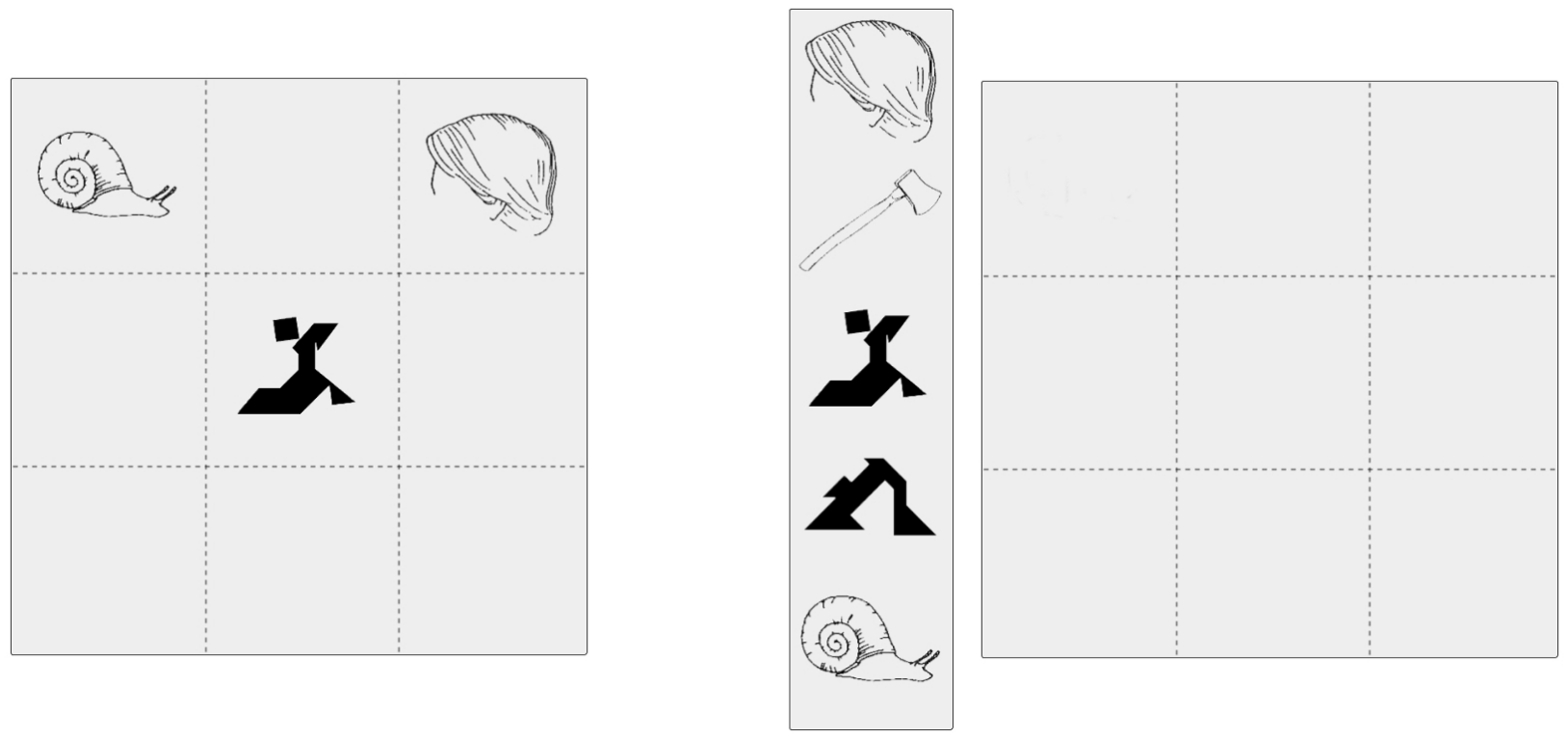
**Example director’s grid (left) and corresponding matcher’s grid (right)**.

The target and filler pictures were inserted into 3 by 3 grids. For each grid, we created a director’s version and a corresponding matcher’s version (see **Figure 1**). The director’s version showed target and/or filler pictures in three of the grid’s nine cells. The matcher’s grids showed nine empty cells. To the left of these empty cells, the matcher’s grids showed a box with five pictures. Three of these pictures were identical to the target and/or filler pictures in the corresponding director’s grid. The other two (irrelevant) pictures were line drawings and tangram shapes that differed sufficiently (visually and semantically) from the target and/or filler pictures. We created two lists (List 1 and List 2), each with sixteen director’s and sixteen corresponding matcher’s grids. All experimental pictures occurred twice in each list, either in successive grids (short lag between interlocutor mention and alternative name) or with two grids intervening (long lag between interlocutor mention and alternative name). Lags were chosen so that each participant would name each experimental picture. Experimental pictures that were in the short lag condition in List 1 were in the long lag condition in List 2, and vice versa.

A female native speaker produced the audio recordings for the current study. The speaker made recordings of both selected names for target pictures (e.g., *die Haare* and *die Frisur, die Jacke* and *das Hemd*, etc., see **Table 1**) and of the strongly preferred name for filler pictures [e.g., *die Schnecke* (*the snail*), *die Rakete* (*the rocket*), etc.]. In addition, the speaker recorded the carrier phrases *Jetzt kommt…* (singular, *Next is…,* literally *Now comes…*) and *Jetzt kommen…* (plural, *Next are…,* literally *Now come…*) and the sentence *Und jetzt kommt die dritte Figur* (*And next is the third figure*, literally *And now comes the third figure*). The carrier phrases and the recordings of the names were concatenated in Praat (Boersma and Weenink, 2014) to create sentences, e.g., *Jetzt kommen die Haare* (*Next is the hair*) or *Jetzt kommt die Frisur* (*Next is the haircut*).

The recorded sentences were combined with the pictures in Lists 1 and 2 as follows: in each grid, two pictures (the ones to be placed first and second in each round) were combined with a recording that named the picture, e.g., *Next is the hair*, while one picture (the one to be placed third, i.e., last) was combined with the recording that did not name the picture, e.g., *And next is the third figure*. All experimental pictures were combined with a recording that named the picture the first time the picture occurred. Half the experimental pictures were combined with a recording that named the picture the second time it occurred. In this case, both occurrences of the pictures were combined with recordings that provided *different* names for the pictures. For example, if a picture was first combined with the recording *Next is the hair*, then the picture was later combined with the recording *Next is the haircut*, and vice versa (alternative name condition). The other half of the experimental pictures were combined with a recording that did not name the picture the second time it occurred (*Next is the third figure*, no alternative name condition). In addition, experimental pictures that were combined with audio recordings that named the pictures the second time they occurred (e.g., *Next is the hair*) in List 1 were combined with audio recordings that did not name the pictures the second time they occurred in List 2 (e.g., *Next is the third figure*), and vice versa.

We then created two versions of each of the two lists of pictures, for a total of four experimental lists (Lists 1A, 1B, 2A, and 2B). The B lists differ from the A lists in the recordings that are associated with experimental pictures and that name the pictures. For example, the first occurrence of the picture paired with the recording *Next is the hair* in the A list was paired with the recording *Next is the haircut* in the corresponding B list. To summarize, the above-described materials yielded a 2 (alternative name) by 2 (picture type) by 2 (interlocutor mention – alternative name lag) design with four experimental lists and a total of 32 experimental and 16 filler pictures distributed over 16 grids.

### PROCEDURE

Two participants played 16 rounds of a pick-and-place game, preceded by two practice rounds. Each round consisted of three trials for a total of 48 trials. One participant was assigned the role of director and one participant was assigned the role of matcher. After each round, participants switched roles. During each round, the director saw a 3 by 3 grid that showed the positions of three objects/shapes (see **Figure 1**). The matcher saw an empty 3 by 3 grid and five objects/shapes to the left of the grid. Participants were told that the director saw the positions for three objects/shapes in his/her 3 by 3 grid and should inform the matcher about the objects’ positions. Furthermore, directors were encouraged to assign a name to the objects/shapes whose position they described. Matchers should position the correct objects/shapes based on the director’s instructions. We emphasized that it was important that the objects were positioned in the correct order and that the director would therefore be informed over headphones about which object was to be positioned first and second (e.g., *Next is the hair*). After the first two objects had been positioned, there was only one object left to be positioned in a given round. It was therefore obvious to the director which object needed to be positioned third and s/he would receive the general instruction *Next is the third figure*. Note that this was not obvious to the matcher, who saw a total of five objects/shapes on the screen and thus another three potential objects/shapes that could still be positioned. Thus, the director needed to name the third object for the matcher and could not just say something like *the last object*. Experimental pictures were distributed across rounds such that they were named by one participant on their first occurrence and by the other participant on the second occurrence. Each session was audio recorded. To summarize, during any given trial, the director heard over headphones which object/shape the matcher should position, then described the position of the object/shape to the matcher, and the matcher placed the object in the described position.

After the main experiment, participants performed working memory and picture naming tests. In particular, participants performed the Wechsler Digit Span backward test (Wechsler, 2008), where they repeated sequences of numbers of increasing length backward. Participants received 1 point for each sequence repeated correctly backward. The test was stopped if two sequences of the same length were not repeated correctly. They also performed a speeded E-Prime (Schneider et al., 2002) version of the Boston Naming test (Segal, 2001). A speeded version was used to avoid ceiling effects in picture naming. Participants were shown a picture for one second and had to name the object in the picture within the one second that the picture was displayed. There were 60 pictures in total and participants received 1 point for each correctly named picture. Finally, participants filled in a questionnaire that provided demographic information about participants and gauged how well participants knew each other. Level of acquaintance was gauged using the following scheme: 0 = not acquainted; 1 = acquainted; 2 = friend or partner.

### DATA CODING

To extract data from the main experiment, the directors’ referring expressions for experimental trials were transcribed. From these transcriptions, we determined which names directors gave to objects/shapes. Most commonly, directors did actually assign names to the objects/shapes, for example, *Frisur (haircut)*. However, directors sometimes provided a more detailed description, for example, *abstrakte Kerze, hat so ein bisschen was vom Anker* (*abstract candle, a little bit like an anchor*). In these cases, at least two different coders decided on a name, in this case, *Kerze* (*candle*). For each first occurrence of an experimental picture, we then coded whether or not the name that the director used was the same as the name s/he heard over headphones. For each second occurrence of an experimental picture, we coded whether or not the name that the director used was the same as the name that the director’s partner (i.e., the interlocutor) had used previously to refer to the object/shape.

## RESULTS

The total data set includes 640 first-occurrence and 640 second-occurrence experimental picture descriptions. The second-occurrence picture descriptions are of interest for our analyses: here, the director named the picture after his or her interlocutor had previously named the picture. The second-occurrence descriptions will therefore be called target trials. We excluded some target trials due to participants’ behavior during first-occurrence picture descriptions: in particular, we excluded 14 (2%) target trials because the director used two different names to describe the object/shape when it *first* occurred, for example, the director described a picture as both *Frisur* (*haircut*) and *Haarschopf* (*head of hair*). We then checked how often directors used the word they heard over headphones to describe the picture the first time it occurred. Participants used the word they heard over headphones in 598 (93%) cases. In the remaining 28 cases (4%), participants used another name, most commonly the alternative name, e.g., *Haare* (*hair*) when *Frisur* (*haircut*) was presented over headphones, and vice versa. Target trials from these 28 cases were also excluded because in the majority of these cases participants would hear the same name from both their interlocutor and the audio recording, and we were interested in which name participants would choose when their interlocutor had used a *different* name than what was presented over headphones. Thus, results from 598 target trials were used for the analysis.

**FIGURE 2:**
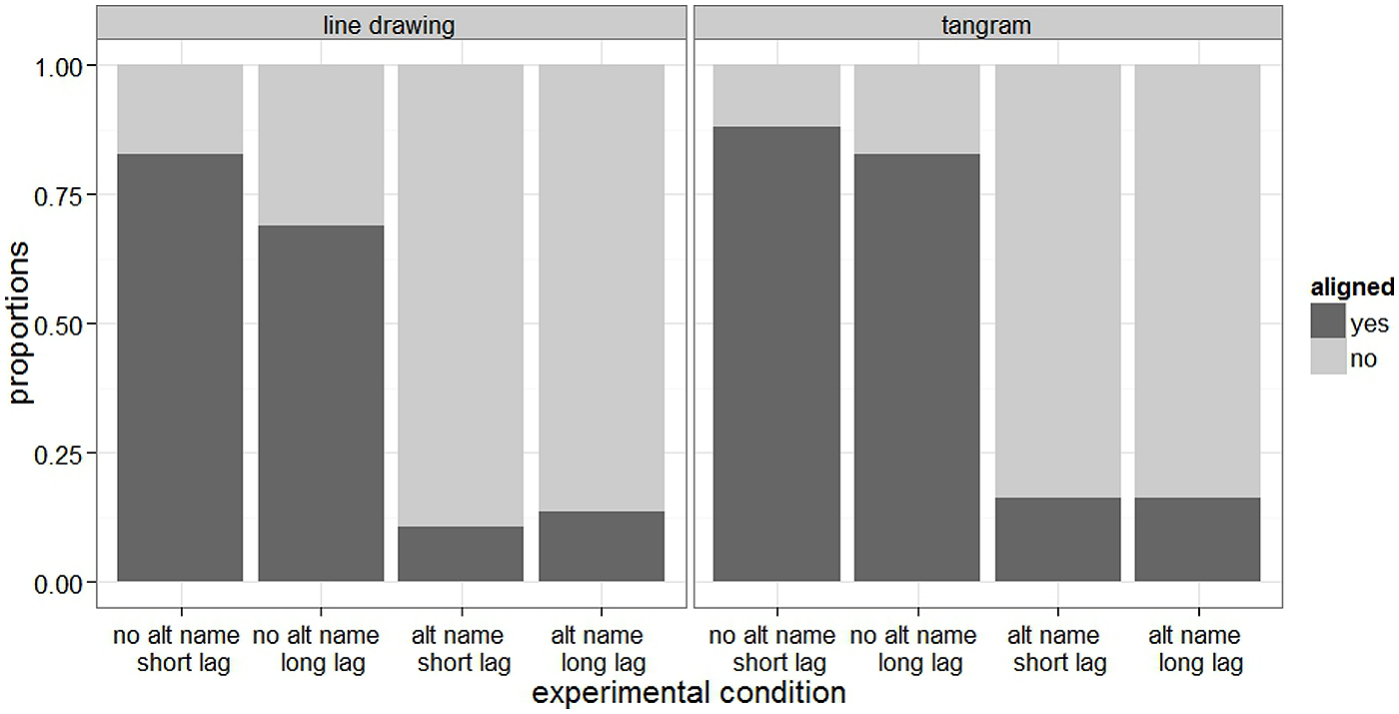
**Proportion of aligned responses, i.e., responses where the director used the name that the partner had used previously use to describe the same object/shape, in the two alternative name (alternative name vs. no alternative name) and in the two lag (short vs. long) conditions for line drawings and tangram shapes**.

**Figure 2** shows the proportions of responses where the director aligned with their partner, i.e., where the director used the name that the partner had previously used to describe the same object/shape. The figure shows that directors aligned quite strongly with their interlocutor when they heard no alternative name, especially when there was a short lag between when the interlocutor had named the picture and when the director named it. In contrast, alignment with the interlocutor was very low when directors heard an alternative name. Overall, tangram shapes seem to elicit slightly more aligned descriptions than line drawings.

The descriptive results from our additional measures showed that participants were able to repeat between 2 and 7 number sequences backward in the Digit Span task, with a mean of 4.35 (SD = 1.29). In the speeded Boston Naming task, participants correctly named between 30 and 58 pictures, with a mean of 45.86 (SD = 5.10). The questionnaire revealed that the vast majority of participants either did not know each other (coded as 0) or were friends or partners (coded as 2). That is, very few participants were merely acquainted (coded as 1).

In the main analysis, we tested which experimental conditions and additional measures affected whether or not directors referred to the objects/shapes with the name that their interlocutor had used previously. We performed mixed-logit analyses (Jaeger, 2008), using the lme4 package (Bates and Sarkar, 2007) of the open source statistical computing and graphics software R (R Development Core Team, 2008). Mixed-logit models are appropriate for the analysis of binomial response variables and they allow modeling random subjects and item effects within the same analysis.

The initial statistical model had alignment (yes vs. no) as response variable. The fixed factors in the initial model were: alternative name (no vs. yes), picture type (line drawing vs. tangram shape), lag (short vs. long), working memory (raw scores from the digit span backward test), word retrieval (raw scores from the speeded Boston Naming test), level of acquaintance (0 = not acquainted; 1 = acquainted; 2 = friend or partner), and all two-way interactions involving the experimental conditions. All fixed factors were centered (to avoid collinearity) and sum coded (for ANOVA-style main effects). The initial model also included participants and items as random effects. Redundant factors and interactions were removed from the initial model. Random slopes were added if they improved model fit (cf. Barr et al., 2013). Model comparisons established the least complex model that provided an equally good fit of the data than more complex models. This final model was considered minimally optimized. The final model included alternative name, picture type, level of acquaintance, and the alternative name by lag interaction as fixed effects. Lag, working memory capacity, word naming ability, and the remaining two-way interactions were redundant fixed factors (as established by model comparisons) and removed from the initial model. The results from the final model are summarized in **Table 2**.

**Table 2:** Results from the final statistical model.

Fixed effect	Estimate	z-value	*p*-value
Alternative name	–3.69400	–4.386	<0.0001
Picture type	0.39441	2.502	<0.05
Level of acquaintance	0.07394	0.409	=0.68
Alternative name by lag interaction	–0.36835	–2.328	<0.05

The analysis reveals a large effect of alternative name, such that participants aligned reliably more with their interlocutor when they heard no alternative name compared to when they heard an alternative name. A look at the raw data revealed that whenever participants did not align with their actual interlocutor after hearing an alternative name, they always used the alternative name provided by the voice over headphones. In addition, there was a significant effect of picture type such that participants aligned reliably more with their interlocutor when naming tangram shapes compared to when naming line drawings. Level of acquaintance, though included in the final model, did not reliably affect alignment. Finally, there was a significant alternative name by lag interaction. To explore the interaction, we fit separate models for the alternative name and no alternative name conditions. The results showed a marginal effect of lag in the no alternative name conditions (estimate = 0.3378, *z* = 1.909, *p* = 0.06; in addition, there was a main effect of picture type with estimate = 0. 3509, *z* = 1.985, *p* < 0.05), but no effect of lag in the alternative prime conditions (no fixed effects in the final model).

## DISCUSSION

This study investigated several factors that may affect the strength of lexical alignment to a specific conversation partner. Replicating previous findings, participants aligned rather strongly with their interlocutor when they did not hear an alternative term over headphones. But when they heard an alternative term, they showed greatly reduced alignment with their actual interlocutor. Instead, they aligned with the voice over headphones. Thus, a word presented over headphones by a voice that gives instructions, but that is not directly addressed in the collaborative task can affect alignment with the actual interlocutor quite substantially. This result has several possible interpretations and implications and we discuss this result both in terms of disrupting alignment with the matcher and establishing alignment with the voice over headphones.

First, this result reveals that there are limits to a partner-specific account in that there may be situations (here, one that is experimentally manipulated) in which interlocutors do not align with the person they are addressing and with whom they are performing a collaborative task. In particular, our situation presented directors with an alternative lexical item provided by a voice over headphones. This alternative lexical item may either have disrupted alignment with the actual interlocutor or caused directors to align with the voice over headphones.

If we view this result in terms of disrupting alignment with the actual interlocutor, then it is compatible with both the idea that alignment is based on a low-level automatic priming process and that alignment is egocentric, or at least not addressee-specific. In particular, the alternative term could have served as a prime that increased the probability that the alternative term would be used and thus interfered with the potential alignment with the interlocutor. Due to its recency, the term presented over headphones was also a convenient choice for participants: they had just heard the term and did not need to come up with their own description or remember what their interlocutor had said. An egocentric processing approach would predict such behavior.

Notice that such disruption had few consequences in the current experimental design: there were only a handful of cases where participants selected an incorrect picture. In fact, the experiment was designed such that the risk of communicative breakdown was very low. All of the pictures shown in the matchers’ grids differed sufficiently from each other, both visually and semantically. Thus, for picture selection it did not matter whether participants referred to a picture as *Haare* (*hair*) or *Frisur* (*haircut*) – there was no other picture in that round that could possibly be referred to with *Haare* or *Frisur*. Participants may have assessed the risk of communicative breakdown and considered it to be low enough to allow them to use the alternative terms.

If we view this result in terms of establishing alignment with the voice over headphones, then it would be compatible with a strategic account of alignment. In particular, directors may have chosen to align with the voice over headphones for several reasons. For example, participants may have taken the voice over headphones to be an expert or authority figure in the experimental context. After all, the voice had privileged information, namely, information about the order in which objects were to be placed. It is thus possible that participants also assigned importance to the particular lexical item that this voice produced. In particular, they could have assumed that the voice over headphones provided them with the ‘correct’ name to describe the picture. This interpretation would be in line with Pirie (2010), who found marginally more lexical alignment to an interlocutor assumed to be a professor compared to one that is assumed to be a fellow student.

However, listener-centered approaches to alignment assume that responses are designed for the addressee, in this case, the actual interlocutor. Thus, an explanation that assumes that directors established alignment with the voice over headphones would need to stipulate reasons for why directors did not align with the person with whom they were performing a collaborative task and to whom they were addressing their utterances, but rather chose to use the lexical items proposed by the voice over headphones. One possibility is that directors felt compelled to use the ‘correct’ terms to describe pictures. Thus, our data could be reconciled with an addressee-centered account by assuming that interlocutors may misalign if they believe that their lexical item is the ‘correct’ one in the current context and they may want to ‘teach’ their interlocutor the correct term in the current context.

In addition to the strong effect of the alternative name, the experiment revealed that interlocutors aligned more when describing tangram shapes than when describing line drawings, regardless of whether or not they received an alternative name. This result points to a strategic, addressee-centered process. In particular, the type of picture affected alignment not only when no alternative name was presented, but also when an alternative name was presented. It seems that participants adapted their choice of label to the difficulty of the task and the accompanying risk of miscommunication: our rating study showed that there were many more possibilities to label the tangram shapes than to label the line drawings. Thus, the tangram shapes were more ambiguous and could be interpreted in more ways. Participants aligned more with their interlocutor in the case of tangram shapes, where there was more ambiguity, than in the case of line drawings, which were less ambiguous. This behavior thus seems to reflect a strategic process that takes the addressee’s needs into account.

Finally, the experiment showed an interaction between the presentation of the alternative name and the lag between the interlocutor’s and the voice over headphones’ referring expressions for the same item. In the no alternative name conditions, we replicate previous findings (cf. Branigan et al., 2011) in that a longer lag between successive mentions of an item (marginally) decreased lexical alignment compared to a shorter lag. In contrast to the no alternative name conditions, we found no lag effects in the alternative name conditions. Thus, when a recent alternative name is presented, the name seems to ‘override’ the original name irrespective of how recently the original name has been encountered.

This study revealed no effect of word retrieval, working memory, or level of acquaintance on the magnitude of alignment. To our knowledge, no previous study has tested if and how word retrieval abilities may affect lexical alignment. Our study did not find an effect. Previous work on working memory capacity suggested that it may influence alignment, in particular, with respect to syntactic alignment (cf. Hartsuiker and Kolk, 1998; Foltz et al., 2014). We thus speculated that working memory capacity may also affect lexical alignment. This does not seem to be the case with this type of experimental design. It is possible that working memory capacity affects syntactic alignment, but not lexical alignment. In this context, it should be noted that Foltz et al. (2014) measured alignment for a syntactic structure that was more complex than the alternative structure. In contrast, our lexical alternatives were equally complex. It is thus possible that working memory capacity modulates the ability to align to complex structures when a simpler alternative is available. Finally, we measured level of acquaintance due to an informal observation that participants who knew each other behaved differently. In addition, previous studies had shown that level of acquaintance modulated attitude alignment (Davis and Rusbult, 2001) and the extent to which participants behaved egocentrically (Savitsky et al., 2011). However, we found no effect of level of acquaintance on lexical alignment.

In sum, while all of our experimental manipulations concerning the communicative situation (alternative name, lag, and picture type) affected alignment magnitude, interlocutors’ individual differences (word retrieval and working memory) and social aspects (level of acquaintance) had no effect. Overall, this pattern of results suggests that adults with different abilities align lexically in a quite similar way, regardless of how well they know their interlocutor. What seems to affect the strength of lexical alignment are the specifics of the communicative situation that interlocutors find themselves in. Further studies would be needed to confirm the pattern we observe here.

Taken together, our results show that if the risk of miscommunication is low, as in our task, then lexical alignment to a specific interlocutor is rather fragile. Our data suggest that alignment has both addressee- and non-addressee-centered components and that neither an interactive alignment approach, nor egocentric processing, nor a conceptual pacts approach can fully account for the data. We propose that any account of the mechanisms behind alignment needs to involve both addressee- and non-addressee-centered processes (cf. Branigan et al., 2011; Behnel et al., 2013).

Our results therefore highlight the need for a hybrid theory that explains how addressee- and non-addressee-centered processes work together during lexical alignment. We argue that interlocutors are quickly able to assess a communicative situation and the need for alignment within this situation. If the communicative situation allows, for example, when there is a low risk of miscommunication, participants may show more non-addressee-centered behavior and less addressee-centered or partner-specific behavior than in situations, where the risk of miscommunication is higher. But when the communicative situation involves greater risk of miscommunication or possibly when social factors, such as status, are involved, participants rely more on partner-specific behavior.

Note that in many communicative situations non-addressee-centered and addressee-centered behavior would lead to similar alignment magnitudes. For example, communicative situations don’t typically involve an auditory ‘diverter’ similar to our experimental manipulation. More typically, the lexical item that an interlocutor used for a certain concept is also the most recently heard lexical item for that concept. Thus, both non-addressee-centered and addressee-centered behavior would lead to the adoption of that lexical item to refer to that concept.

The current study suggests further research in at least two directions. Further research is needed to disentangle the mechanisms and processes that lexical alignment relies on in different communicative situations. In addition, further studies are needed to determine in more detail how non-addressee-centered behavior and addressee-centered behavior interact in lexical alignment and which communicative situations encourage which behaviors. Further studies with more natural conversational settings are also needed. Our study did not involve a typical communicative situation. Studies involving three or more naïve interlocutors may provide a more natural alternative to our experimental design. We propose that pitting two lexical alternatives against each other and observing which lexical choices interlocutors make during conversation is a fruitful approach to investigate the mechanisms behind the alignment phenomenon and to what extent these mechanisms are or are not addressee-centered.

## Conflict of Interest Statement

The authors declare that the research was conducted in the absence of any commercial or financial relationships that could be construed as a potential conflict of interest.
